# Oxidative Stress in Oral Diseases: Understanding Its Relation with Other Systemic Diseases

**DOI:** 10.3389/fphys.2017.00693

**Published:** 2017-09-14

**Authors:** Jaya Kumar, Seong Lin Teoh, Srijit Das, Pasuk Mahakknaukrauh

**Affiliations:** ^1^Department of Physiology, Universiti Kebangsaan Malaysia Medical Centre Kuala Lumpur, Malaysia; ^2^Department of Anatomy, Universiti Kebangsaan Malaysia Medical Centre Kuala Lumpur, Malaysia; ^3^Forensic Osteology Research, Chiang Mai University Chiang Mai, Thailand; ^4^Excellence in Osteology Research and Training Center, Chiang Mai University Chiang Mai, Thailand; ^5^Department of Anatomy, Faculty of Medicine, Chiang Mai University Chiang Mai, Thailand

**Keywords:** oral, disease, oxidative stress, pathways, free radicals, inflammation

## Abstract

Oxidative stress occurs in diabetes, various cancers, liver diseases, stroke, rheumatoid arthritis, chronic inflammation, and other degenerative diseases related to the nervous system. The free radicals have deleterious effect on various organs of the body. This is due to lipid peroxidation and irreversible protein modification that leads to cellular apoptosis or programmed cell death. During recent years, there is a rise in the oral diseases related to oxidative stress. Oxidative stress in oral disease is related to other systemic diseases in the body such as periodontitis, cardiovascular, pancreatic, gastric, and liver diseases. In the present review, we discuss the various pathways that mediate oxidative cellular damage. Numerous pathways mediate oxidative cellular damage and these include caspase pathway, PERK/NRF2 pathway, NADPH oxidase 4 pathways and JNK/mitogen-activated protein (MAP) kinase pathway. We also discuss the role of inflammatory markers, lipid peroxidation, and role of oxygen species linked to oxidative stress. Knowledge of different pathways, role of inflammatory markers, and importance of low-density lipoprotein, fibrinogen, creatinine, nitric oxide, nitrates, and highly sensitive C-reactive proteins may be helpful in understanding the pathogenesis and plan better treatment for oral diseases which involve oxidative stress.

## Oxidative stress and related diseases in the body

Oxidative stress occurs as a state of disturbance between free radical produced and the capability of antioxidant system to counteract such (Pisoschi and Pop, 2015). Free radicals are also classified as reactive oxygen species (ROS) or reactive nitrogen species (RNS) and both possess unpaired valence electrons. ROS can be further classified into oxygen centered radicals (superoxide anion, hydroxyl radicals, alkoxyl radicals, and peroxyl radicals) and oxygen centered non-radicals (hydrogen peroxide and singlet oxygen), while RNS consists of nitric oxide, nitric dioxide and peroxynitrite (El-Bahr, 2013). ROS are naturally occurring oxidants involved in numerous cellular biochemical events that are essential to life but at the same time capable of causing harmful oxidative stress when overproduced (McCord, 2000). Free radicals cause damage to all essential biocompounds such as DNA, proteins, and membrane lipids, thereby causing cause cell death. These free radicals are countered by inherent antioxidant system that exists in two major groups: enzymatic (glutathione peroxidase, myeloperoxidase, superoxide dismutase, and catalase) and non-enzymatic (minerals, vitamins, polyphenols, and thiols; Gilgun-Sherki et al., 2001; Pisoschi and Pop, 2015).

Oxidative stress forms the basis of cancer, diabetes, rheumatoid arthritis, non-alcoholic fatty liver disease, chronic inflammation, stroke, aging, and numerous neurodegenerative diseases (Fridovich, 1999; Fang et al., 2002; Gentric et al., 2015; Pisoschi and Pop, 2015). Different epidemiological and clinical studies showed evidence of important role of oxidative stress and impairment of antioxidant defense systems in the pathogenesis, neoangiogenesis, and dissemination of local or distant cancers, such as cancers of the ovary and prostate (Oh et al., 2016; Saed et al., 2017). In any cancer, oxidative stress induced by hypoxia, was reported to promote oncogenic protein (MUC4) degradation via autophagy, enhancing the survival of cancer cells in the pancreas (Joshi et al., 2016). In Parkinson's disease, oxidative stress and aggregation of protein are the key pathogenic processes, where aggregation of α-synuclein results in aberrant free radical production and neuronal death (Deas et al., 2016). Similarly in Alzheimer's disease, amyloid-β inserts into the membrane systems to begin the oxidative stress during the disease progression in the brain (Swomley and Butterfield, 2015). In addition, increased oxidative stress and reduced superoxide dismutase levels were observed in human peripheral blood mononuclear cells which were obtained from patients with mild cognitive impairment (Mota et al., 2015).

## Oxidative stress related to oral diseases

Oral diseases such as periodontitis, dental caries, cancer in the oral cavity, HIV/AIDS, diseases involving mucosal and salivary glands, orofacial pain, and clefts, affect the oral health and hygiene (Jin et al., 2016). Global Burden of Disease 2015 study showed individuals with untreated oral conditions to increase from 2.5 billion in the year 1990 to 3.5 billion in 2015, with a 64% increase in disability-adjusted life year (Kassebaum et al., 2017). In addition, the direct and indirect treatment expenses due to dental diseases worldwide, were approximately US$442 billion in 2010 (Listl et al., 2015).

Among all oral diseases, the periodontal disease (comprising gingivitis and periodontitis), accounted for 3.5 million years lived with disability, US$54 billion/year in lost productivity and a major portion of the US$442 billion/year cost for oral diseases (Tonetti et al., 2017). Oxidative stress was involved in the progression of periodontitis, a chronic inflammatory disease of the periodontal tissue, caused by disturbance in the regulation of the host inflammatory in response to bacterial infection (Kataoka et al., 2016; Kanzaki et al., 2017). In chronic periodontitis, there was lower serum total antioxidant level and salivary capacity when compared to the control individuals (Ahmadi-Motamayel et al., 2017). Biomarkers of lipid peroxidation (one of the oxidative stress-mediated pathways) such as 8-isoprostane and malondialdehyde (MDA) were high in patients affected by chronic periondontitis (Akalin et al., 2007; Matthews et al., 2007; Pradeep et al., 2013). In addition, assessment of blood and gingival tissues of chronic periodontitis patients also revealed mitochondrial DNA deletion (5 kbp; Canakci et al., 2006). Gingival blood analysis of periodontitis patients also marked high level of 7-8-dihydro-8-ossiguanina (8-oxoG), a pre-mutagen base that results from ROS-mediated DNA damage (Takane et al., 2002; Krol, 2004). Similarly, higher level of 8-isoprostane concentration (an alternative approach to estimate lipid peroxidation) in the crevicular fluid of gingiva was detected in chronic periodontitis patients compared to those with gingivitis and healthy individuals (Pradeep et al., 2013). Serum reactive oxygen metabolite levels in periodontitis patients positively correlated to antibody levels with regard to bacteria such as *Porphyromonas gingivalis, Prevotella intermedia*, and *Eikenella corrodens* (Tamaki et al., 2014). Following scaling and root planning after systemic antioxidant lycopene administration, there was decrease in the oxidative stress and improvement in clinical parameters, which was maintained up to 4 months after discontinuation of antioxidant treatment (Ambati et al., 2017).

The pathogenesis of chronic inflammatory disease like oral lichen planus (OLP) is not well-understood (Tvarijonaviciute et al., 2017). Various studies showed that oxidative stress is involved in the pathogenesis of OLP. Significantly higher salivary ROS, lipid peroxidation, nitric oxide, and nitrite levels were found in OLP patients compared to the control subjects (Batu et al., 2016; Mehdipour et al., 2017; Tvarijonaviciute et al., 2017). The total antioxidant activity was significantly decreased in OLP patients with increased level of salivary malondialdehyde (MDA) compared to the healthy control group suggesting the possible role of the oxidants to orchestrate the disease via lipid peroxidation-mediated pathway (Lopez-Jornet et al., 2014; Shiva and Arab, 2016).

Oxidative stress was also correlated with oral cancer, as increased lipid peroxidation and reduced antioxidants was reported in patients suffering from stage II, III, and IV oral cancer (Manoharan et al., 2005). In addition to these findings, nitric oxide-mediated DNA damage was reported in patients with oral leukoplakia. Samples of oral epithelium taken from these patients recorded high levels of 8-nitroguanine and 8-oxoG (Ma et al., 2006).

## Oxidative stress mediate cellular damage

The deleterious effects of ROS in the event of oxidative stress are through lipid peroxidation and irreversible protein modification that leads to cellular apoptosis or programmed cell death (Ferrari, 2000). Numerous pathways mediate oxidative cellular damage and these include as caspase pathway, PERK/NRF2 pathway, NADPH oxidase 4 pathway and JNK/mitogen-activated protein (MAP) kinase pathway.

## Pathways that mediate oxidative cellular damage

### Caspase pathway

Caspases are a family of cysteine protease enzymes that carry out programmed cell death and inflammation. During apoptosis, caspases are activated to ensure that programmed cell death occurs with less damage to nearby tissues and also to ensure the degradation of components of the cell in a well-controlled manner (Rathore et al., 2015). Functionally, the caspases are classified into two major groups in apoptosis: Initiator caspases such as caspase-8 and -9, activate downstream caspases known as the executioner. These include caspase-3, -6, and -7 which are responsible for the breakdown of the cellular proteins (Creagh and Martin, 2001). In chronic periodontitis, caspase-3 concentration was significantly increased in gingival crevicular fluid and serum, and significantly correlated to the probing depth, gingival index, and clinical attachment level, thereby indicating apoptosis plays an important role in the destruction of periodontium tissues in chronic periodontitis (Pradeep et al., 2016).

There are two major caspase-associated apoptotic pathways related to oxidative damage (1) mitochondrial mediated pathways, and (2) the death receptor mediated pathway. In cells, caspases exist as zymogens (pro-caspases) which are activated only in the presence of appropriate stimulus such as the insult of oxidative stress (Slee et al., 1999). During activation, the caspases undergo proteolytic cleaving to dimerize into an active enzyme (Alnemri et al., 1996). In the mitochondria-mediated apoptotic pathway, hydrogen peroxide (H_2_O_2_) releases cytochrome C which binds to the apoptotic protease activating-factor 1 (Apaf-1) to initiate caspase-9 activation (Madesh and Hajnoczky, 2001; Andoh et al., 2002). ROS-mediated oxidative modification of caspase-9 at C403 residue promotes the interaction of caspase-9 with Apaf-1 via disulfide bonding that results in the apoptosome formation which leads to activation of caspase-9 (Zuo et al., 2009). The executioner, caspase-3 is speculated to be the converging point in both mitochondria-dependent and independent pathways in oxidative stress-driven apoptosis (Ueda et al., 2002; Kanthasamy et al., 2003). H_2_O_2_ activation of caspase-3 lead to activation of PKC delta and this contributes to the nuclear DNA breakdown and apoptotic cell death (Carvour et al., 2008).

**Figure 1:**
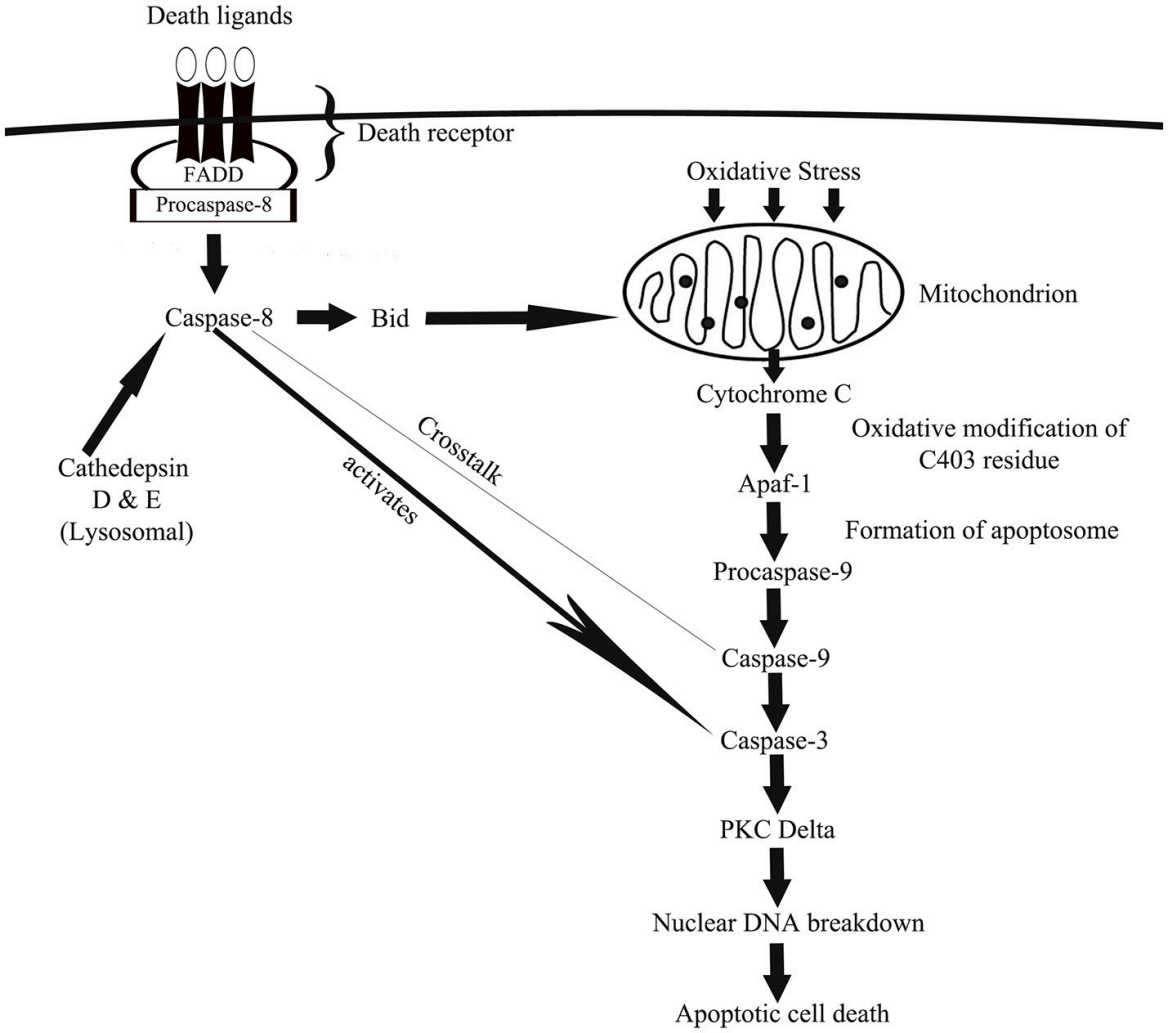
A schematic depiction of how caspase-associated pathways leads to oxidative damage via mitochondrial- and death receptor-mediated cellular apoptosis. In mitochondrial-mediated apoptosis, generation of ROS causes the release of cytochrome C from mitochondria, which via a cascade of cellular actions activates caspase-9 and then caspase-3 that eventually channels cell death. In death receptor-mediated apoptosis, oxidative stress-driven death ligands activates caspase-8, which then activates a pro-apoptotic protein known as Bid, which once activated travels to mitochondria to facilitate mitochondria-mediated apoptotic pathway. Death receptor-activated caspase-8 also activates caspase-3 to induce cell death.

In death receptor-mediated apoptotic pathway, caspase-8 channels apoptosis following oxidative stress (Baumgartner et al., 2007). Death receptors (also known as death-domain receptors) can promote the cleavage of pro-caspase-8 with appropriate stimulation (Boldin et al., 1996). Then, caspase-8 activates downstream executioner caspases (caspase-3; Jiang and Wang, 2004) or cleaves a pro-apoptotic protein known as Bid, which once activated translocates to the mitochondria and causes the release of cytochrome C, followed by fragmentation of DNA and apoptosis (Li et al., 1998). Interestingly, numerous studies reported crosstalk between caspase-8 and caspase-9 (Figure 1; Basu et al., 2006; Mareninova et al., 2006). Using pancreatic acinar cells, Baumgartner et al. (2007) showed reported partial inhibition of caspase-8 activation by caspase-9 and vice versa during H_2_O_2_-mediated apoptosis. The same study also reported the involvement of lysosomal proteins such as cathedpsin D and E (aspartyl proteases from lysosomes) in activation of caspase-8.

### NADPH oxidase 4 (NOX4) pathway

NADPH oxidases (NOX) are enzymes that are known to catalyze the electron transfer from NADPH to molecular oxygen, to generate ROS as a microcode in immune response (DeLeo and Quinn, 1996). NOX protein family consists of NOX1, -2, -3, and -4 (Sahoo et al., 2016). The NOX4 isoform is expressed everywhere in the body, including heart, neuron, kidney, liver, and endothelial cells (Byrne et al., 2003; Vallet et al., 2005; Ray et al., 2011; Babelova et al., 2012; Crosas-Molist et al., 2014). NOX4 predominantly generates H_2_O_2_ in mitochondria where it is usually localized (Ago et al., 2008; Nisimoto et al., 2014; Sanders et al., 2015).

**Figure 2:**
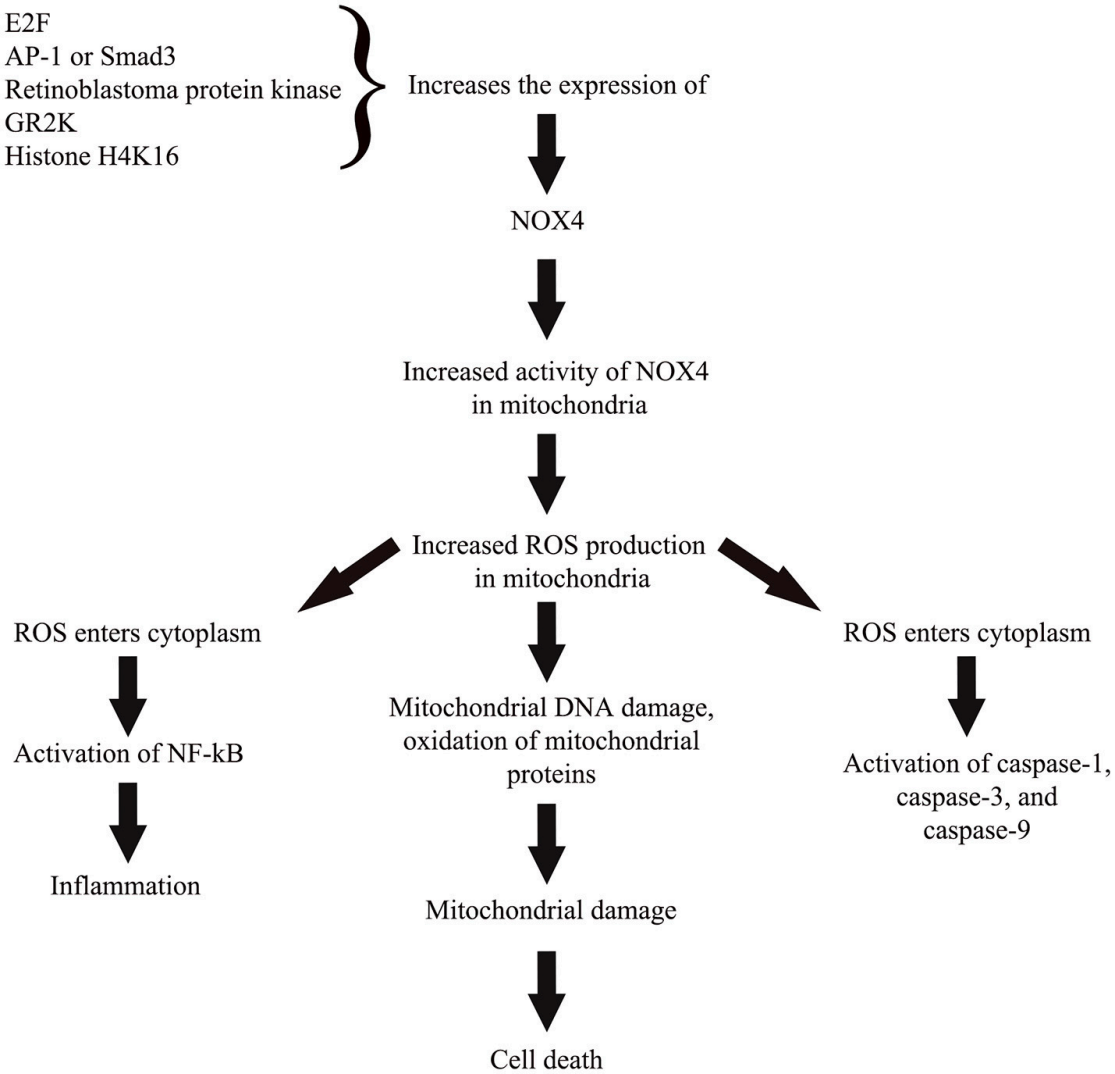
A simplified representation on the role of NOX4 in cellular pathway toward ROS-induced oxidative stress. Activity of NOX4 is regulated through regulation of the enzyme's expression by various transcription factors, protein kinase, cellular receptor, and epigenetic regulator. Enhanced NOX4 expression or activity in mitochondria leads to increased ROS production that subsequently causes mitochondrial damage and cell death. Excess ROS also likely to travel toward cytoplasm and activate numerous pro-apoptotic proteins and also activate nuclear factor kappa beta to trigger a pro-inflammatory state of the cell.

Recent research highlighted the mechanistic effects of NOX4 in oxidative stress (Vendrov et al., 2015; Theccanat et al., 2016). Unlike other isoforms of NOX, the NOX4 do not require cytosolic regulatory subunits in order to be activated. Instead, the enzyme is regulated by transcription factors which include E2F (Zhang et al., 2008), AP-1/Smad3 complex (Bai et al., 2014), retinoblastoma protein kinase (by regulating the activity of E2F), G-protein coupled receptor kinase 2 (Theccanat et al., 2016), and also via epigenetic regulation through increased association of histone H4K16 (Sanders et al., 2015). Enhanced NOX4 expression/activity and mitochondrial localization positively correlates to ROS production in mitochondria (Vendrov et al., 2015). Increased mitochondrial ROS leads to mitochondrial DNA damage, oxidation of mitochondrial proteins, and eventually apoptotic cell death (Madamanchi and Runge, 2013). This overproduced ROS is also likely to enter cytoplasm and activate numerous pro-apoptotic proteins such as caspase-9 and -3 (Tariq et al., 2013), caspase-1 (Moon et al., 2016). Excess ROS also causes a pro-inflammatory shift in the gene expression through nuclear factor-kappa-β activation (NF-κβ; Ungvari et al., 2007). Significant increased NOX4 levels was observed following inflammatory or hypoxic stimulation in periodontal ligament cells, which was accompanied by up-regulation of ROS and catalase levels (Figure 2; Golz et al., 2014). However, prolonged exposure to both stimuli leads to a decreased in catalase level suggesting the collapse of the antioxidative mechanism favoring oxidative stress and inflammatory response as observed in periodontitis (Golz et al., 2014).

### NRF2—antioxidant response element signaling pathway

The nuclear factor erythroid 2 (NFE2)-related factor (NRF2), a basic leucine zipper (bZIP) protein from the cap “n” collar (CNC) subfamily, protects against oxidative stress. NFE2 regulate the expression of antioxidant and detoxification proteins (Gold et al., 2012). NRF2 plays an important role in numerous diseases such as rheumatoid arthritis, atherosclerosis, oral cancer, and chronic periodontitis (Kim et al., 2010; Huang et al., 2013; Sima et al., 2016). NRF2 plays a role as a positive regulator of human Antioxidant Response Elements (AREs; Venugopal and Jaiswal, 1996). NRF2 was reported to regulate the expression of numerous antioxidant enzymes, which include glutathione peroxidase, glutathione S-transferase, catalase, and superoxide dismutase (Niture et al., 2014).

**Figure 3:**
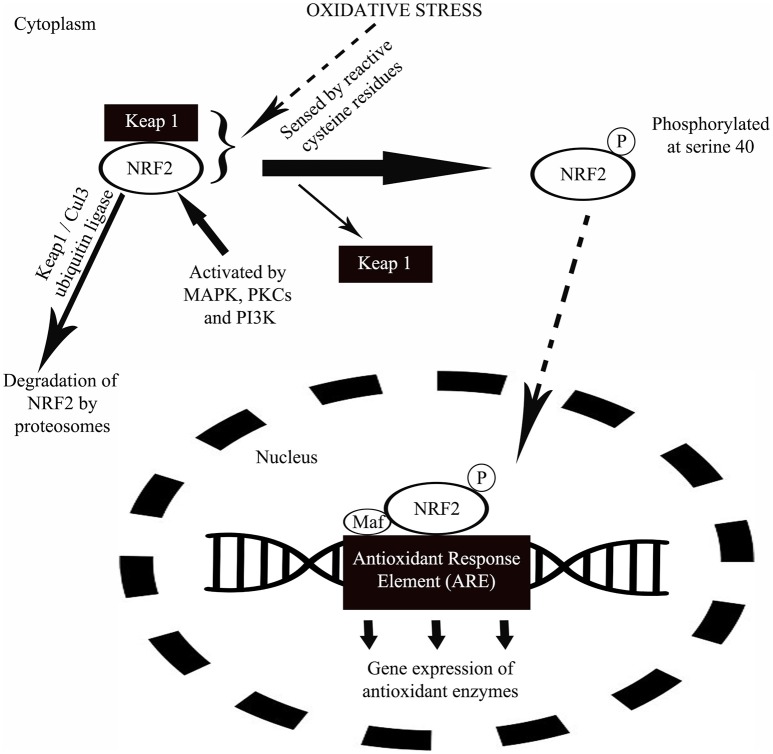
A schematic diagram showing the mechanism of NRF2 in protection against oxidative stress. Under normal condition, a protein called Keap1 suppresses the activity of NRF2 by physically binding to NRF2 and at the same time anchored to cytoplasm. Following a period of inactivity, NRF2 is degraded by proteasomes. During oxidative stress, oxidants will be sensed by the Keap1/NRF2 complex via reactive cysteine residues in Keap1. Upon oxidant sensing, NRF2 undergoes phosphorylation at serine40 releasing Keap1 from the complex. This is followed by the translocation of NRF2 to nucleus where the protein forms a heterodimer with small transcription factor Maf and binds to antioxidant response enzyme (ARE) of numerous antioxidant gene promoter regions to initiate their transcription in response to oxidative stress.

The NRF2 activation was reported to involve a protein known as Kelch-like erythroid cell-derived protein with CNC homology-associated protein 1 (Keap 1) by Itoh et al. (1999). The same study reported that under basal condition, Keap 1 anchored to cytoplasm act as a suppressor of NRF2 by physically binding to NRF2 and prevent the translocation of NRF2 to the nucleus to activate ARE-containing gene promotor regions (Itoh et al., 1999). Under this condition, NRF2 is rapidly degraded by proteasomes (Kobayashi et al., 2004) through polyubiquitination via Keap1/Cul3 ubiquitin ligase (Wakabayashi et al., 2003). Keap1/NRF2 complex “senses” the oxidative stress directly via reactive cysteine residues in Keap1 (Dinkova-Kostova et al., 2004) and NRF2 (Huang et al., 2002). NRF2 is activated by protein kinase C, MAP kinase and phosphatidylinositol-3 kinase (Yu et al., 1999; Kang et al., 2002; Numazawa et al., 2003). Following the “oxidant sensing,” NRF2 is phosphorylated at serine40 to be released from Keap1 (Huang et al., 2002) and translocates to the nucleus where there is formation of a heterodimer with Maf and binds to AREs of numerous antioxidant gene promoter regions to begin their transcription (Figure 3; Itoh et al., 1997).

The critical role of NRF2 in protection against oxidative stress was shown through myriad studies (Chan et al., 2001; Talalay et al., 2003; Motohashi and Yamamoto, 2004). A recent study reported significant down-regulation of NRF2 pathway in patients with severe chronic periodontitis (Sima et al., 2016). NRF2 over-expression significantly improved anti-oxidative levels, increased cell proliferation, and inhibited periodontal ligament stem cell apoptosis (Liu et al., 2017).

### Role of different inflammatory markers

Inflammation and oxidative stress were found to be associated with numerous chronic diseases such as diabetes, cardiovascular disease (CVD), hypertension, alcoholic liver diseases, neurodegenerative diseases, cancer, and aging (Biswas and de Faria, 2007; Cachofeiro et al., 2008; Ambade and Mandrekar, 2012; Tucker et al., 2015). The accepted hypothesis is that inflammation can trigger oxidative stress and the oxidation also can induce inflammation.

Inflammation is the protective response of our biological system toward harmful exogenous and endogenous stimuli (Ferrero-Miliani et al., 2007). Inflammation is also an inherent immune response employed to safeguard our health. However, unregulated or exaggerated prolonged inflammation can cause tissue damage and chronic diseases. During the onset of inflammation, transcription factors such as activating protein-1 (AP-1) and NF-κβ induce pro-inflammatory gene expressions, which increases production of ROS while inducing oxidative stress (Tabas and Glass, 2013). Certain ROS such as H_2_O_2_ can enhance the pro-inflammatory gene expression (Flohe et al., 1997) through activation of numerous cellular pathways. Oxidative stress activates NOD-like receptor protein 3 (NRLP3) inflammasome (Shimada et al., 2012) which are responsible for maturation of pro-inflammatory proteins such as interleukin (IL)-18 and IL-1β (Schroder and Tschopp, 2010). The expression of pro-IL-1β was upregulated in human oral squamous cell carcinoma tumors and it increased the proliferation of dysplasia oral cells, stimulated oncogenic cytokines, and promoted the severity of oral squamous cell carcinoma (Lee et al., 2015).

### Role of oxygen species signal assembly of the NLRP3 inflammasome

The inflammasome is a part of the innate immune system and it responds to microbes or cellular stress through regulation of caspase-1 activation and induction of inflammation (Lamkanfi and Dixit, 2009). Among the numerous NLR inflammasome complexes such as NLRP1, -P2, -P3, -P6, -P12, and -C4 (Correa et al., 2012; Allen et al., 2013; Chen, 2014), the NLRP3 inflammasome influences the chronic inflammation and maturation of pro-inflammatory IL-1β and IL-18 (Davis et al., 2011). The expression of NLRP3 was significantly higher in patients with chronic periodontitis and generalized aggressive periodontitis, which were mainly distributed in inflammatory cells (Xue et al., 2015; Ran et al., 2017). *P. gingivalis* infection increased loss of alveolar bone, production of IL-1β, IL-6, IL-18, gingival gene expression of pro-IL-18 and pro-IL-1β, and activity of caspase-1 in peritoneal macrophages of wild-type mice, unlike in NLRP3-deficient mice. This suggests that *P. gingivalis* activate innate immune cells through the NLRP3 inflammasome in periodontal disease (Yamaguchi et al., 2017). Similarly, *Fusobacterium nucleatum* infection involving the gingival epithelial cells, leads to NLRP3 inflammasome-dependent secretion of IL-1β (Bui et al., 2016). Activation of NLRP3 causes the activation of caspase-1 which is integral for the maturation of IL-18 and IL-1β into active cytokines and also the initiation of pryoptosis (Lamkanfi, 2011; Zhao et al., 2016).

Improper regulation of inflammasome could lead to the imbalance in between pro- and anti-inflammatory cytokines and result in inflammation and pryoptosis. NLRP3 inflammasome was reported to be activated by a host of molecules such as excess ROS, glucose, ATP, ceramides, sphingosine, crystals of cholesterol, uric acid, and oxidized LDL (Duewell et al., 2010; Jiang et al., 2012; Luheshi et al., 2012; Bandyopadhyay et al., 2013; Fukumoto et al., 2013). The exact underlying molecular mechanisms that regulate the assembly and activation of NLRP3 were not fully elucidated. However, recent studies reported the ROS signaling to activate NLRP3 inflammasome (Fukumoto et al., 2013; Heid et al., 2013).

The cellular source of ROS in influencing the activation of NLRP3 inflammasome arises from the byproduct of mitochondrial oxidative phosphorylation, NOX, xanthine oxidase, cyclooxygenase, and lipooxygenase (Habu et al., 1990; Lacy et al., 1998; Andrew and Mayer, 1999; Paravicini and Touyz, 2008; Sorbara and Girardin, 2011; Heid et al., 2013). For mitochondrial-ROS dependent NRLP3 inflammasome activation, numerous molecules such as saturated fatty acid palmitate (Wen et al., 2011), liposome (Zhong et al., 2013), and mitochondrial cardiolipin (Iyer et al., 2013) were reported to be involved. NOX acts as a mediator in various molecules associated activation of NRLP3 complex. For an instance, excess of extracellular ATP binding to P2X7 receptors in mammals leads to rapid accumulation of ROS that eventually activate NRLP3 inflammasome (Riteau et al., 2012). The origin of ROS caused by excess ATP is reported to be NOX-derived (Cruz et al., 2007). Akin to ATP, alum, particulated metals and uric acid crystals were also shown to activate NLRP3 inflammasome via NOX-driven ROS generation (Martinon, 2010).

### Lipid peroxidation as a result of infection

Lipid peroxidation (LPO) is the oxidative deterioration of lipids caused by ROS. LPO is a chain reaction that mostly affects polyunsaturated fatty acids due to the presence of methylene bridges (-CH_2_-) that possess reactive hydrogen atoms (Halliwell and Gutteridge, 1984). The chain reaction consists of three major steps including initiation, propagation and termination. For an in-depth information on the mechanism of LPO, we would suggest the readers to refer to review written by Repetto et al. (2012). The end-products of LPO are aldehyde, ethane, pentane, 2,3-transconjugated diens, isoprostains, and chlesteroloxides (Ustinova and Riabinin, 2003).

LPO has been implicated in numerous non-communicable diseases and aging-related disorders such as cataract, rheumatoid arthritis, atherosclerosis, and neurodegenerative diseases (Niki et al., 2005). In addition to these ailments, LPO was linked to infections such as influenza virus (Mileva et al., 2000; Kumar et al., 2003), acute and chronic fascioliasis (Kaya et al., 2007) and *Helicobacter pylori* infection (Davi et al., 2005). In addition, LPO as shown by salivary MDA level, was significantly increased in patients suffering from chronic periodontitis, OLP, oral leukoplakia, and oral squamous cell carcinoma (Baltacioglu et al., 2014; Malik et al., 2014; Metgud and Bajaj, 2014; Shirzad et al., 2014; Trivedi et al., 2015).

### Role of polyphenols

Polyphenols are naturally occurring compounds that are found in vegetables, fruits, beverages, herbs and spices. Examples of polyphenols include isoflavones, flavanols, flavones, phenolic acids, resveratrol, tannins, curcumin, anthovyanidins, and lignans (Tanigawa et al., 2007). In plants, polyphenols provide front line of protection from pathogens and ultraviolet light (Pandey and Rizvi, 2009).

Recent advances in research focusing on the anti-inflammatory and antioxidant effects of the polyphenols have shed light on the mechanisms of the phenolic compounds in scavenging free radicals, regulation of cytokine activities, and the maintenance of antioxidant enzyme system. Phenolic compounds scavenge free radicals through donation of an electron or hydrogen atom to various reactive oxygen, chlorine and nitrogen species (Tsao and Li, 2012). Phenolic compounds also directly inhibit Fe^3+^ reduction and thus generate reactive OH· (Perron and Brumaghim, 2009). These free radical scavenging and metal chelating effects of phenolic compounds interrupts the propagation stage of the LPO. Dietary phenolic compounds are able to restore inherent antioxidant enzymatic activities such as the superoxide dismutase, glutathione peroxidase, catalase, and glutathione reductase. Phenolic compounds control the expression of these enzymes through regulation of transcription factor NRF2 activities which in turn influences the ARE-mediated expression of the mentioned enzymes (Kohle and Bock, 2006). Flavanols, isoflavones, and flavones were reported to regulate the transcriptional activities of NRF2 (Zhang et al., 2003; Kohle and Bock, 2006).

In addition to antioxidant effects, dietary phenolic compounds were also shown to possess protective effects on inflammation through modulation of NLRP3 inflammasome. Recently, Hori et al. (2013) showed that green propolis rich in cinnamic acids inhibited inflammasome mediated secretion of IL-1β and activation of caspase-1. In separate studies, flavonoids such as procyanidin B2 and apigenin inhibited inflammasome-mediated secretion of IL-1β in LPS-induced human macrophages (Zhang et al., 2014; Martinez-Micaelo et al., 2015). Dietary phenolic compounds also reduced inflammation by attenuating pro-inflammatory cytokine-induced activation of NF-κβ by acting as AhR agonist regulator. By doing such, phenolic compounds modulate AhR-mediated signaling pathways that are involved in the activation of NF-κβ (Kohle and Bock, 2006; Vogel et al., 2014).

## How oral infections are linked to other diseases

Oral health is an important aspect of overall well-being of an organism. Numerous systemic conditions and diseases have oral origins (Beck et al., 1996; Li et al., 2000). At oral cavity, saliva act as the first line of defense against free radicals (Amerongen and Veerman, 2002; Battino et al., 2002) through antioxidants such as catalase, superoxide dismutase, and glutathione peroxidase (Battino et al., 2002). In the event of an infection, increased generation of free radicals outnumber antioxidants to initiate oxidative stress.

### Periodontitis and circulating oxidants

Geerts et al. (2002) assessed the level of endotoxins in blood (pro-inflammatory factors) following mastication in patients with periodontitis. Endotoxin level was significantly higher following mastication in patients with severe periodontitis, thereby suggesting the possible detrimental effect of the oral disease on systemic health (Geerts et al., 2002). Myriad of clinical and pre-clinical findings were reported periodontal inflammation-generated ROS to diffuse into bloodstream, and gradually affecting other organs (Sobaniec and Sobaniec-Lotowska, 2000; Tomofuji et al., 2007; Baltacioglu et al., 2014). In addition to oxidants, the level of circulatory antioxidants were reported to be lower in periodontitis patients (Baltacioglu et al., 2006; Konopka et al., 2007).

Smoking is regarded as one of the most significant risk factors for the development of periodontitis. Smoking can also increase oxidative stress. Smoking may affect the alveola and tooth loss may be a feature. Smoking is perhaps the only modifiable cause which can check periodontitis.

### Cardiovascular disease

**Figure 4:**
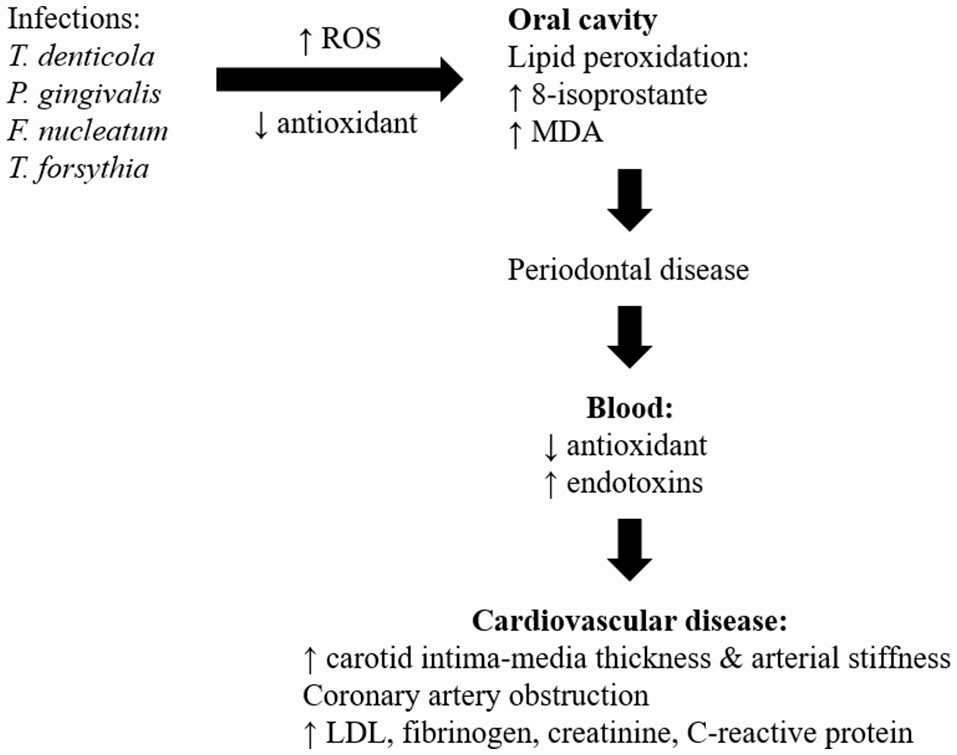
Schematic diagram to show how oral disease may be linked to cardiovascular disease.

Numerous cross-sectional studies and systematic reviews highlighted oral diseases, particularly periodontitis which could be a risk factor for development of atherosclerotic CVD (Ahn et al., 2016; Bengtsson et al., 2016; Berlin-Broner et al., 2016; Gomes et al., 2016; Hansen et al., 2016; Khatri et al., 2016; Zeng et al., 2016; Beukers et al., 2017; Natarajan and Midhun, 2017). A cross-sectional analytical study showed an association between periodontitis and dental parameters (gingival recession, pocket depth, clinical attachment level, and bleeding on probing) with the severity of coronary artery obstruction being measured by angiography (Ketabi et al., 2016). Similarly, periodontitis was associated with increased thickness of carotid intima-media and arterial stiffness, which are indicators of subclinical atherosclerosis and predicts CVD risk (Houcken et al., 2016; Wu et al., 2016). However, studies also showed lack of significant association between periodontal variables and obstruction of coronary vessels (Zanella et al., 2016). The risk of a myocardial infarction for the first time and peripheral arterial disease was significantly increased in patients with periodontitis (Ryden et al., 2016; Calapkorur et al., 2017). The link between periodontal disease and CVD with respect of detailed clinical findings in the patient was summarized in Table 1. The role of different microorganisms, involvement of ROS, different mechanisms involved, inflammatory markers, and the development of CVD was also represented in Figure 4.

**Table 1:** Association between periodontal disease and cardiovascular diseases.

**Participants**	**Sample size**	**Findings**	**References**
Adults with aged >40 years in Korea.	1,343	Periodontitis was associated with subclinical atherosclerosis and peripheral arterial disease.	Ahn et al., 2016
Adults with aged 60–96 years in Sweden.	499	Significant association between periodontitis and carotid calcification.	Bengtsson et al., 2016
Participants in the baltimore longitudinal study of aging.	278	Periodontal disease, endodontic burden, number of teeth and oral inflammatory burden were associated with incident cardiovascular events.	Gomes et al., 2016
Periodontitis patients in Denmark	100,694	Periodontitis patients were at higher risk of myocardial infarction, ischemic stroke, cardiovascular death, major adverse cardiovascular events, and all-cause mortality.	Hansen et al., 2016
Dental patients in University of Amsterdam.	109	Periodontitis is associated with increased arterial stiffness.	Houcken et al., 2016
Coronary artery obstruction patients in Isfahan, Iran.	82	Positive correlation between variables gingival recession, pocket depth, clinical attachment level, decayed, missing, decayed-missing-filled, bleeding on probing, and degree of coronary artery obstruction.	Ketabi et al., 2016
Adults with aged 35–65 years in Bhopal, India.	40	Periodontitis patients was associated with higher carotid intima-media thickness and diastolic blood pressure.	Khatri et al., 2016
Adults with mean age 46 years, mean BMI 21.1 kg/m2 in Bangladesh.	917	Mean attachment loss was associated with increased carotid intima-media thickness.	Wu et al., 2016
Patients that underwent coronary angiography.	195	No significant associations were found between periodontal variables and vessel obstruction. Tooth loss was found to be a risk indicator for coronary heart disease.	Zanella et al., 2016
Meta-analysis of 15 observational studies	17,330	Presence of periodontal disease was associated with carotid atherosclerosis.	Zeng et al., 2016
Dental patients aged >35 years in Netherlands.	60,174	Periodontitis showed significant association with atherosclerosis.	Beukers et al., 2017
Patients referred from the Department of Cardiovascular Surgery to Department of Periodontology.	60	Periodontitis raised the odds ratio for having peripheral arterial disease.	Calapkorur et al., 2017
Adults with aged 20–40 years in India.	60	Severe generalized periodontitis was associated with subclinical atherosclerosis	Natarajan and Midhun, 2017

The main mechanisms linking oral diseases to CVD involve actions of oral bacteria on the blood vessels, and systemic inflammation (Kholy et al., 2015). Bacterial (*T. denticola, P. gingivalis, F. nucleatum*, and *T. forsythia*) genomic DNA were detected in visceras such as aorta, heart, kidney, lung, and liver in mice induced with periodontitis suggesting that oral bacteria were able to gain entry into the blood stream from inflamed gingival thereby producing low-level transient bacteremia (Chukkapalli et al., 2017). Various periodontal bacteria were identified in atherosclerotic plaques and were deemed possible contributors to the disease. Different bacterial co-occurrences were detected in the plaques of subgingiva and plaque of atherosclerosis patients, including *T. forsythia, T. denticol, P. gingivalis* and *P. nigrescens* (Mahalakshmi et al., 2017). The bacteria in the atherosclerotic plaques created biofilms that stimulated the innate immune system reactant Toll-like receptor 2. This contributed to chronic inflammation and continued the immune system activation (Allen et al., 2016). Study also showed positive correlation between the periodontal bacteria levels and CVD risk associated mediators (low-density lipoprotein, fibrinogen, creatinine, and highly sensitive C-reactive proteins) levels in subjects with periodontitis and atherosclerosis (Bozoglan et al., 2017).

The local production and accumulation of inflammatory mediators in severe generalized periodontitis can cause systemic inflammation and endothelial dysfunction (Tonetti, 2009). *P. gingivalis* oral infection in mice, induced alterations in systemic cytokine production, i.e., up-regulation of matrix metalloproteinase 3, intercellular adhesion molecule 1, insulin-like growth factor binding protein 2, chemokine (C-X-C motif) ligand 7 and the down-regulation of interleukin-17, L-selectin, and tumor necrosis factor-α (Miyauchi et al., 2012). Similarly in patients with coronary heart disease, periodontitis was associated with increased systemic inflammation (elevated IFN-γ, IL-10, and TNF-α levels; Kampits et al., 2016).

Recent studies indicated that periodontal treatment attenuated pro-artherosclerotic factors. Following periodontal treatment, white blood cells, low-density lipoprotein, platelet, fibrinogen, creatinine, and highly sensitive C-reactive proteins levels were significantly reduced and high-density lipoprotein levels significantly increased in patients with periodontitis and atherosclerosis, as well as in patients diagnosed with periodontitis alone (Bozoglan et al., 2017). Similarly, systemic markers of atherosclerosis: adrenomedullin and chemokine (C-C motif) ligand 28 levels changed significantly in the periodontitis and atherosclerotic patients, compared to the non-artherosclerotic periodontitis patients following non-surgical periodontal treatment. Decrease in serum neopterin induced by periodontal treatment contributed to the increased arterial elasticity in periodontitis patients (Ren et al., 2015). The results of these studies suggests that removal and reduction of these periodontal bacteria in subgingival plaque may be an important prophylactic measure to periodontitis and atherosclerosis (Mahalakshmi et al., 2017).

### Liver diseases

Using rat model of periodontitis (lipopolysaccharide/protease-induced), Tomofuji et al. (2008) reported oxidative DNA damage in the liver of experimental rats. Supporting the notion, another rat model of periodontitis, that is the ligature-induced model showed a decrease in the liver antioxidant, glutathione and increase in circulating level of hexanoyl-lysine suggesting a possible link between periodontitis-generated oxidants and liver damage (Tomofuji et al., 2008). In humans, limited literature has related periodontitis and liver diseases (Furuta et al., 2010; Han et al., 2016).

### Pancreatic disease

A recently published meta-analysis associated periodontitis with pancreatic cancer. The study estimated the relative risk for periodontitis and pancreatic cancer to be 1.74 (95% Cl, 1.41–2.15) based on findings from three continents (Maisonneuve et al., 2017). Despite a large number of studies associating periodontitis and pancreatic, however, the underlying mechanism of the disease is poorly understood.

### Gastric disorders

*H. pylori* (bacteria implicated in gastritis and peptic ulcers) has been shown to be harbored by periodontal pockets in periodontitis patients (Soory, 2010). In parallel to this finding, periodontitis patients showed presence of *H. pylori* in subgingival biofilm (Riggio and Lennon, 1999; Gebara et al., 2004). Gastric carcinoma has been correlated with *H. pylori* infection-mediated ROS production, DNA damage along other endogenous and exogenous factors (Farinati et al., 2008). In a study investigating the effects of outer membrane vesicles of *H. pylori* in human gastric epithelial cells, oxidative stress-associated genomic damage with glutathione was noticed (Chitcholtan et al., 2008). In a separate study, *H. pylori* elicited mitochondrial damage in gastric epithelial cells, causing oxidative burst and mitochondrial-ROS mediated apoptosis (Calvino-Fernandez et al., 2008).

### Alzheimer's disease

Studies have suggested significant association between periodontitis and Alzheimer's disease, which may be mediated through effects on systemic inflammation (Ide et al., 2016; Leira et al., 2017). The risk of developing dementia were higher for periodontitis patients aged 65 and older, compared to healthy individuals (Shin et al., 2016; Lee et al., 2017). Alzheimer's patients showed high serum IL-6 levels while periodontitis patients had high serum TNF-α levels (Cestari et al., 2016). The association between these cytokine levels in periodontitis and Alzheimer's patients suggests their implication in the overlapping mechanisms between periodontitis and Alzheimer's disease (Cestari et al., 2016). In addition, patients with severe periodontitis had higher blood Aβ_1−42_ levels and higher Aβ_42/40_ ratio (Gil-Montoya et al., 2017).

## Summary and perspective

Oxidative stress causes damage to various organs in the human body. Proper understanding of oxidative stress and its pathways, free radicals and inflammatory markers related to oral diseases are important for effective treatment. Future drug targets may be planned according to the different pathways involved in inflammation and oxidative process.

## Author contributions

Conceptual framework and design: SD, PM. Searched references: JK and SLT. Drafted manuscript: SD, JK, and SLT. Critically revised the manuscript: SD, JK, SLT, PM.

### Conflict of interest statement

The authors declare that the research was conducted in the absence of any commercial or financial relationships that could be construed as a potential conflict of interest.
